# Cases from the Dispensary Practice of Pennsylvania College

**Published:** 1851-03

**Authors:** H. J. Richards

**Affiliations:** Clerk of the Medical and Surgical dispensary


*Cases from the dispensary practice of Pennsylvania College. Reported by Mr.* H. J. Richards, *Clerk of the Medical and Surgical dispensary.*

It cannot be expected that in the ordinary routine of a Collegiate dispensary practice, in a field so well gleaned as this, that many cases occur of sufficient interest to merit the attention of the practical community. If this were so, it would materially impair the usefulness of the institution, as an elementary system of practical instruction; as this object can only be obtained by presenting to a class the most ordinary and frequent forms of disease that we are called upon to relieve.

Nevertheless, the register of a given number of cases cannot fail to offer some pages of sufficient importance to the profession to justify their presentation. The cases hereto annexed, have been selected chiefly with reference to some special points of interest, which it is believed they possess. Case 2d, deserves attention from its rare occurrence.

The slimness of this, our first report, is accounted for by the statement, that previously to the present session, the proceedings of the Dispensary had not been recorded, and by the omission of the cases of operative surgery. The latter, embracing some peculiar plastic operations, have been reserved for the next month's report.

The duties of the medical dispensary are discharged by the Professor of the chair of Practice—Dr. Darrach. The prescribing day is Saturday, at 3½ o'clock, P. M.

*Case* 1.—A sequelum of scarlatina simplex in the form of a chronic superficial ulceration, extending over the entire anterior surface of the leg in a girl of 13 years of age. Successfully treated by elevated position of the limb, cold absorbent poultices encased externally in silk oil cloth, and subsequently by dry rye meal.

The child had had an attack of scarlet fever, in Ohio, in 1843, at the age of 6 years. As a sequelum on the first week, a subdermal fibrinous transudation occurred, and as its consequence the above mentioned cutaneous affection. This on examination was found to consist of a chronic hyperemia of the chorion, studded with innumerable pin-hole ulcerations of the derm. Its existence has been seven years, inducing chronic œdema of the limb, with general indisposition, and hazarding chlorosis at the appropriate revolutionary period of life.

The patient was confined to the recumbent position—the left lower limb elevated, and the ulcer covered with a cold absorbent poultice externally cased in silk oil cloth. In two weeks the minute ulcerations healed, the abnormal exaltation of temperature and consequent distress ceased, and the redness lessened. In order to establish an epidermis, the dry application of rye meal was substituted for the cool, moist dressing, and after another fortnight, she was perfectly recovered.

The basis of the difficulty in the case was, most likely, struma; and had cod liver oil been at an early period administered, the distressing chronic hyperemia and ulceration would not probably have occurred.

*Case 2nd.*—W—— L——; presented, Dec. 21st, 1850, æt. 21, pedlar. Spare habit, lymphatic temperament; has been of intemperate habits; pulse and functions of the digestive tube natural; cutaneous exhalation deficient; breathing laborious, attended with pain in the side after exertion; mottled reddish brown discoloration of the skin upon the anterior and posterior aspects of the trunk, extending partly over the upper extremities, and prolonged over the groin and thighs. The color in some places was uniform—at others it presented isolated patches of dead, white skin, in striking contrast with the liver-colored deposit. The white islet-like patches are the results of the total destruction of the pigmentary substance, in the second stage of the disease. Are not the instances occasionally reported of the metamorphoses of negroes into whites, precisely similar in character; the gradual and irregular bleaching process being exhibited in greater contrast upon the dark skin of the former?

This complaint is figured in Mr. Wilson's splendid illustrations of cutaneous diseases, recently published, as chloasma or liver-spot. It is a diseased condition of the chromatogenous function resulting in an *alteration* of the pigmentary deposit; this serving to distinguish it from those diseases whose prominent feature is an exaggeration of the natural redness. The particular disease with which it is likely to be confounded, is Pityriasis, and the practitioner is aided in forming this erroneous diagnosis by the unfortunate nomenclature of Willan who has described it as Pityriasis versicolor. In addition to the above distinction, it is remarked by Mr. Wilson that "in chloasma there is no primary desquamation of the epiderma; and when desquamation does occur it is *mealy*, or foliaceous. In Pityriasis, desquamation is a prominent character; and the separation of the epiderma occurs in furfuraceous scales. In chloasma, again, the elevation of the surface is very slight and the lines of motion are but little deepened; while in Pityriasis, they are strongly marked and sometimes to the extent of producing chapping of the skin and bleeding."

The causes of this disease are obscure. It is scarcely necessary to put in a caution against the error of inferring from the vulgar name an analogous diseased condition of the hepatic function. The prevalent disposition to regard all eruptions of the skin as of syphilitic origin, must be guarded against. It is known to be associated with uterine irritation in women; and will be recognized as not an unusual symptom in pregnancy. In this instance exposure to the sun after bathing was the supposed cause.

The treatment pursued in the present case was the alterative plan, adapted to the vast majority of these cutaneous affections.

Solution of the arsenite of potassa as the general means; a lotion of potassii sulphuretum, as a local alterative. The case is progressing towards a cure.

*Case 3d.*—A healthy looking male child, æt. three years, was presented, suffering under palsy of the lower limbs. This was preceded, as was learned by the child's history, by general paralysis. The various functions of the nutritive organism were unaffected; appetite usual, nutrition normal, growth unimpeded, and no evidence existed of any organic lesion of the spinal cord or ganglionic system. *The urine upon examination presented no evidence of an excess of phosphatic deposits.* The lower extremities had their full contour, although from disease they had become flaccid. The affection could not be traced to any former complaint of sufficient gravity to rank as a probable cause. It was therefore treated with entire success as a case of spinal adynamia. The ammoniated copper, gr. v., one hour after each meal, at first provoked nausea, but tolerance having been established, its tonic and alterative property was most satisfactorily manifested.

*Case* 4.—Margaret R———, æt. 2 years, with an affection of six months standing, manifesting the following alarming symptoms—cataleptoid spasms unaccompanied with pain, lasting about five minutes, occurring about once in the month, during which time respiration is almost entirely suspended. In the intervals the child is irritable, sleepless, and liable to a seizure from exposure to any unusual influence, as fright or emotions of a lighter kind. Nothing could be discovered to shed light upon the pathology of this singular affection, involved in the cimmerean obscurity of this class of nervous diseases. An interesting question suggested by the late views of Dr. Marshall Hall, upon the diastaltic nervous system, arises here. Are children who survive these severe forms of convulsive affection, peculiarly apt in remote years to become the subjects of epileptoid affections?

The case was entered as one of spinal irritation, affecting especially the respiratory tract. Absolute rest, and exclusion of any unusual excitement, with attention to the bowels, were enjoined.

